# Pure Anatase Phase Titanium Dioxide Films Prepared by Mist Chemical Vapor Deposition

**DOI:** 10.3390/nano8100827

**Published:** 2018-10-13

**Authors:** Qiang Zhang, Chaoyang Li

**Affiliations:** 1 School of Systems Engineering, Kochi University of Technology, Kami, Kochi 782-8502, Japan; 216005z@gs.kochi-tech.ac.jp; 2 Center for Nanotechnology, Kochi University of Technology, Kami, Kochi 782-8502, Japan

**Keywords:** titanium dioxide, anatase, thin films, mist chemical vapor deposition, growth control

## Abstract

In this research, pure anatase phase titanium dioxide thin films were successfully fabricated for the first time using the mist chemical vapor deposition method, and optional values for deposition temperature and concentration of titanium tetraisopropoxide were established. It was found that the crystallinity of the titanium dioxide film was significantly improved by increasing the deposition temperature. The best crystallinity of titanium dioxide film was obtained at 400 °C. It was confirmed that pure anatase phase titanium dioxide films could be obtained using different concentrations of titanium tetraisopropoxide. The lower concentration of titanium tetraisopropoxide produced better crystallinity in the resultant titanium dioxide film. The morphologies of the titanium dioxide thin films were also significantly influenced by the concentration of titanium tetraisopropoxide in the precursor solution.

## 1. Introduction

Metal oxide gas sensors have been widely applied in various fields. Among the different types of metal oxide gas sensors, metal oxide thin-film gas sensors have attracted considerable interest, as they readily facilitate integration and miniaturization in structure. In addition, such thin-film gas sensors have higher sensitivity and shorter response time than thick film-type metal oxide gas sensors [1,2].

Recently, titanium dioxide (TiO_2_) thin films have been investigated as a promising sensing material for application in various thin-film reducing gas sensors due to their outstanding sensing properties, chemical stability and electrical properties [3,4,5,6].

Among the three different phases of TiO_2_ films, anatase phase TiO_2_ films have better sensitivity to H_2_ and volatile organic compound (VOC) gases than rutile phase TiO_2_ films [7,8]. Hitherto, anatase phase TiO_2_ thin films have been synthesized by various techniques, including magnetron sputtering, high-vacuum chemical vapor deposition (HV-CVD), atomic layer deposition (ALD), electron beam evaporation, and sol-gel method [3,6,9,10,11,12,13,14,15,16,17,18,19,20,21]. However, the TiO_2_ thin films obtained by these methods are mostly a mixture of anatase and rutile phase TiO_2_ [16,17,18,19,20,21]. Although there are some reports on fabricating pure anatase phase TiO_2_ films, their thermal stability is poor [9,19,20] due to the presence of (112) surface in anatase phase TiO_2_ [22,23,24,25,26], which limits the application of anatase phase TiO_2_ films as sensing material.

To fabricate pure anatase phase TiO_2_ film with high thermal stability, it is necessary to develop a novel synthesis method. In our previous research, we used a novel mist chemical vapor deposition method (mist CVD) to synthesize zinc oxide films [27]. Compared with other reported methods, even several publications using similar mist droplets source supply stage [28,29], this novel mist CVD had advantages in terms of precise growth controllability and large area deposition, as well as low cost and simplicity [27,30], and can be expected to be an alternate method to synthesize TiO_2_ thin films. 

There have been many publications on the simulation of binary systems during the CVD process [31,32,33,34,35,36]. Among these works, simulations on the thermal decomposition mechanism of temperature and titanium tetraisopropoxide (TTIP) have been analyzed and reported by some groups [32,33,34]. Reinke et al. [33] investigated the surface reaction kinetics of TTIP in HV-CVD and proposed a comprehensive surface kinetic model, which could be a reference for the simulation of the mist CVD method.

In this research, the mist CVD method was used to synthesize pure anatase phase TiO_2_ thin films. The deposition temperature and solution concentration are significant parameters for mist CVD method, which can greatly influence the properties of TiO_2_ film. Therefore, the effects of deposition temperature and titanium tetraisopropoxide (TTIP) concentration in the solution on the structural properties of TiO_2_ thin films were investigated.

## 2. Materials and Methods 

### 2.1. Introduction of a Novel Mist CVD System

**Figure 1:**
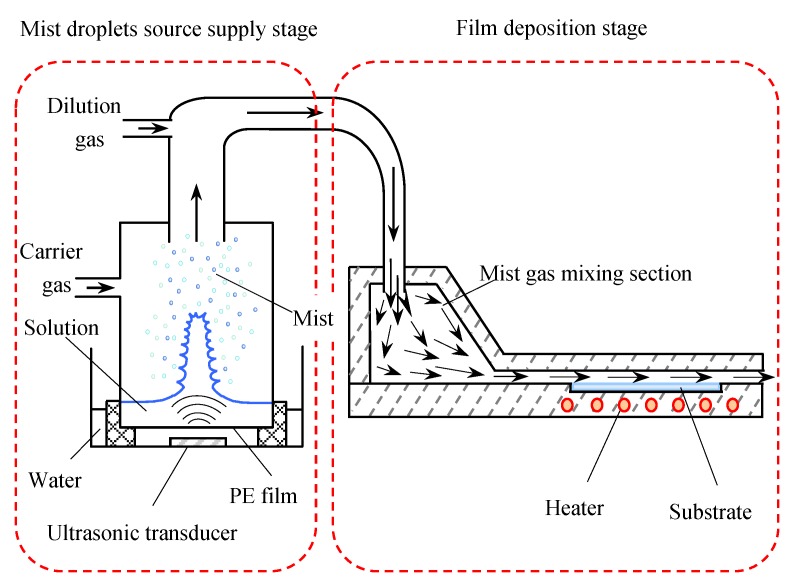
Schematic diagram of fine channel mist chemical vapor deposition (CVD) equipment.

The schematic diagram of the novel mist CVD system is shown in Figure 1. The mist CVD system consisted of two stages: a mist droplet source supply stage and a film deposition stage (in reaction chamber). In the first stage, the solution of precursors was transformed into mist droplets by three ultrasonic transducers, the frequency of which was 2.4 MHz. In the second stage, the mist droplets were firstly transported to a large area referred to as “mist gas mixing section”. Then, the mist droplets were transported into a designed narrow channel structure named the ‘‘fine channel’’. Finally, TiO_2_ thin films were deposited on the substrate, which was set in the fine channel with the appropriate heating [30].

### 2.2. Dependence of Deposition Temperature

The deposition condition is shown in Table 1. TTIP (Wako Pure Chemical Industries, Ltd., Osaka, Japan) was dissolved in ethanol (Wako Pure Chemical Industries, Ltd., Osaka, Japan) to prepare a solution of precursor. Compressed air was used as both a carrier and a dilution gas. The flow rates of the carrier gas and dilution gas were set at 2.5 L/min and 4.5 L/min respectively. Quartz glass was selected as a substrate and set in the reaction chamber. To investigate the influence of deposition temperature, the temperature was set at a range between 200 °C to 400 °C with an interval of 50 °C. 

### 2.3. Dependence of TTIP Concentration

Based on the results of temperature dependence, an optimized temperature of 400 °C was chosen as the deposition temperature for further solution dependence experiments. To investigate the effect of TTIP concentration on structural properties of TiO_2_ films, the TTIP concentration was adjusted from 0.05 mol/L to 0.40 mol/L for comparison, as shown in Table 2. The other deposition parameters including carrier gas, dilution gas and their flow rates were the same as Section 2.2. Before the deposition, the deposition rates at different TTIP concentrations were measured and confirmed. The deposition times at different TTIP concentrations were adjusted to keep the thickness of all TiO_2_ films at 300 nm.

### 2.4. Characterization

The thickness of TiO_2_ thin films were measured by spectroscopic ellipsometry (WVASE32, J.A. Woollam, Co., Inc., Lincoln, CA, USA). The deposition rate was calculated from the thickness of TiO_2_ film and deposition time. The structural properties of TiO_2_ thin films were investigated by using grazing incidence X-ray diffraction (GIXRD, ATX-G, Rigaku, Tokyo, Japan) at a 0.35° incidence angle over a range from 20° to 80°, X-ray photoelectron spectroscopy (XPS, AXIS-HS, Shimadzu/KRATOS, Kyoto, Japan) and Raman spectroscopy (LabRAM HR-800, Horiba Jobin Yvon, Longjumeau, France) with a 532.8 nm excitation laser. The morphologies of thin films were evaluated by atomic force microscope (AFM, Nano-R2, Pacific Nanotechnology, Santa Clara, CA, USA) and field emission scanning electron microscopy (FE-SEM, SU-8020, Hitachi, Tokyo, Japan). To confirm stability of the TiO_2_ films, the adhesion between TiO_2_ film and substrate was checked by ultrasonic oscillation and using adhesive tapes. All of the measurements were carried out at room temperature.

## 3. Results and Discussion

**Figure 2:**
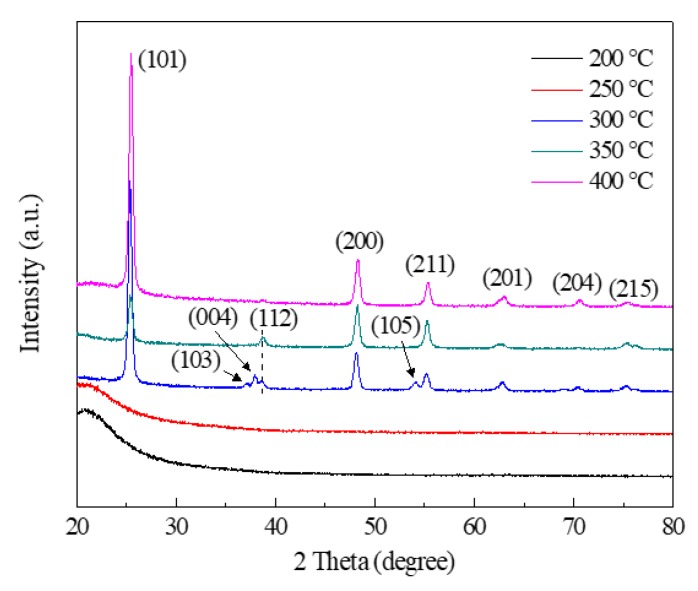
Grazing incidence X-ray diffraction (GIXRD) patterns of TiO_2_ films deposited under different temperatures using the mist CVD method.

Figure 2 shows the GIXRD patterns of TiO_2_ films deposited under different temperatures. There were no obvious peaks observed in the GIXRD patterns of TiO_2_ films deposited at temperatures from 200 °C to 250 °C. The diffraction peaks were identified clearly as the deposition temperature increased from 300 °C to 400 °C, which corresponded with the reflections from (101), (103), (004), (112), (200), (105), (211), (201), (204), and (215) crystal planes of the anatase phase TiO_2_, respectively. It was clear that the (101) peaks were the predominant peaks, indicating that the (101)-oriented anatase facets were dominant for the as-deposed TiO_2_ films during deposition at temperatures from 300 °C to 400 °C. The crystallinity of anatase TiO_2_ film was significantly improved with the increase in temperature. The highest (101) peak intensity was obtained at 400 °C, which suggested that the TiO_2_ films with the best crystallinity were obtained at this temperature.

**Figure 3:**
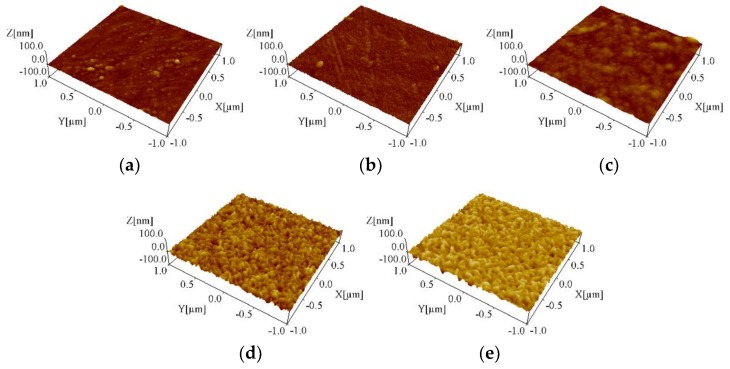
Atomic force microscope (AFM) images of TiO_2_ films deposited under different temperatures using the mist CVD method ((**a**) 200 °C; (**b**) 250 °C; (**c**) 300 °C; (**d**) 350 °C; (**e**) 400 °C).

To confirm the uniformity, the TiO_2_ films deposited under different temperatures were imaged by AFM, as shown in Figure 3. It was clearly observed that the TiO_2_ films fabricated at different temperatures were uniform and flat. The root mean square (RMS) values of the film surface roughness are summarized in Table 3. The roughnesses of all TiO_2_ films were less than 7 nm and showed a tendency to increase as the deposition temperature increased from 200 °C to 400 °C.

During the deposition process, the chemical reactions significantly depended on the temperature. It was reported that the thermal decomposition mechanism of TTIP could be separated as two reactions, including both pyrolysis (Equation (1)) and hydrolysis (Equation (2)) [32].

(1) $$\mathrm{Ti}{\left({\mathrm{OC}}_{3}H_{7}\right)}_{4}\to {\mathrm{TiO}}_{2}+4C_{3}H_{6}+2H_{2}O$$

(2) $$\mathrm{Ti}{\left({\mathrm{OC}}_{3}H_{7}\right)}_{4}+2H_{2}O\to {\mathrm{TiO}}_{2}+4C_{3}H_{7}\mathrm{OH}$$

The required temperature of pyrolysis was higher than 250 °C. The required temperature of hydrolysis was higher than 175 °C in the presence of H_2_O [35].

During the mist CVD process, the pyrolysis of TTIP would not occur when the deposition temperature was lower than 250 °C. The hydrolysis of TTIP (Equation (2)) could not occur at 200 °C, although the reaction temperature was higher than 175 °C, because there was no additional H_2_O participating, which was the reason no peaks were observed in GIXRD patterns for the TiO_2_ film deposited at between 200 °C and 250 °C.

The thermal decomposition mechanism of TTIP in ALD and HV-CVD was analyzed and reported by some groups [32,33,34,35,36,37]. Reinke et al. [33] investigated the surface reaction kinetics of TTIP in HV-CVD and proposed a comprehensive surface kinetic model. Based on the surface kinetic parameters from their model, the reaction rate constant (*k*) under different temperatures can be calculated using the Arrhenius equation (Equation (3)).

(3) $$k=\mathrm{Aexp}(-E_{a}/RT)$$

where A is the frequency factor, E*_a_* the activation energy of reaction, R the universal gas constant and *T* the reaction temperature (in kelvin). 

**Figure 4:**
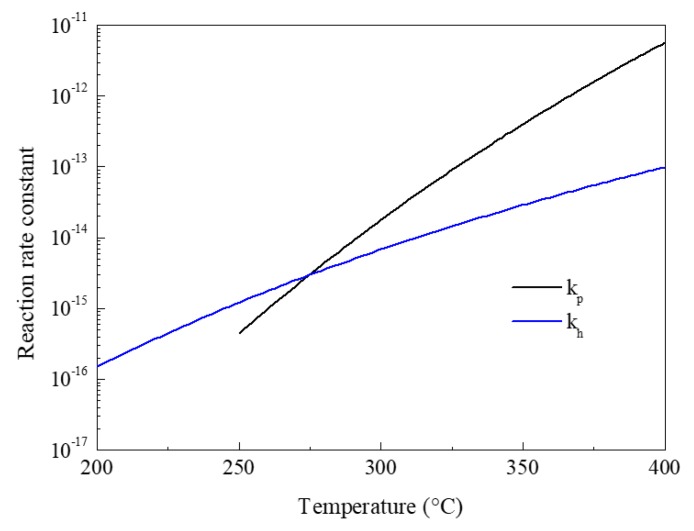
Reaction rate constant of TTIP pyrolysis and hydrolysis reactions under different temperatures.

As shown in Figure 4, the reaction rate constants of hydrolysis (*k_h_*) and pyrolysis (*k_p_*) in the mist CVD processes were calculated using the Arrhenius equation. During the calculation, the activation energies of hydrolysis and pyrolysis were set as 86 kJ/mol and 185 kJ/mol [33]. It was found that both *k_h_* and *k_p_* increased with an increase in temperature. When the deposition temperature was 250 °C, *k_p_* was lower than *k_h_*, because the frequency factor of pyrolysis was much lower than that of hydrolysis. Because the activation energy of pyrolysis was higher than that of hydrolysis, *k_p_* showed a faster growth speed than that of *k_h_*. When the temperature was lower than 274.56 °C, *k_h_* was higher than *k_p_*. When the temperature was greater than 274.56 °C, *k_p_* was higher than *k_h_*, which suggested that the TiO_2_ films deposited from 300 °C to 400 °C could be obtained by the pyrolysis of TTIP.

**Figure 5:**
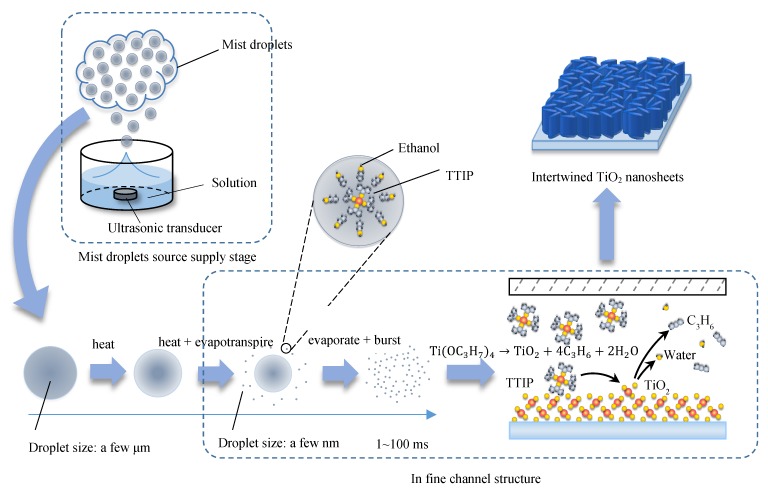
Mechanism of TiO_2_ films deposition during the mist CVD process.

Based on the above calculation and relevant research [30], we propose a model (as shown in Figure 5) to describe the mechanism of TiO_2_ films deposition using the mist CVD process. In the mist droplets source supply stage, the mist droplets—including both TTIP and ethanol—were transformed from the solution of precursors by ultrasonic transducers. Following that, the mist droplets were transported into mist gas mixing section by carrier and dilution gases. During the transportation of mist droplets in the reaction chamber, the average size of mist droplets decreased from a few micrometers to a few nanometers under the influence of heat, evapotranspiration and burst. The mist droplets could maintain the nanoscale in the area of fine channel. Due to gravity and absorption, the mist droplets moved effectively onto the substrate which was set on the heating plate. In this experiment, when the deposition temperature was set from 300 °C to 400 °C, TTIP was decomposed due to the pyrolysis reaction resulting in the generation of anatase phase TiO_2_ films.

To investigate the effect of TTIP concentration in solution of precursor on the structural properties of the TiO_2_ thin films, the optimized temperature of 400 °C was selected for further investigation on TTIP concentration dependence.

**Figure 6:**
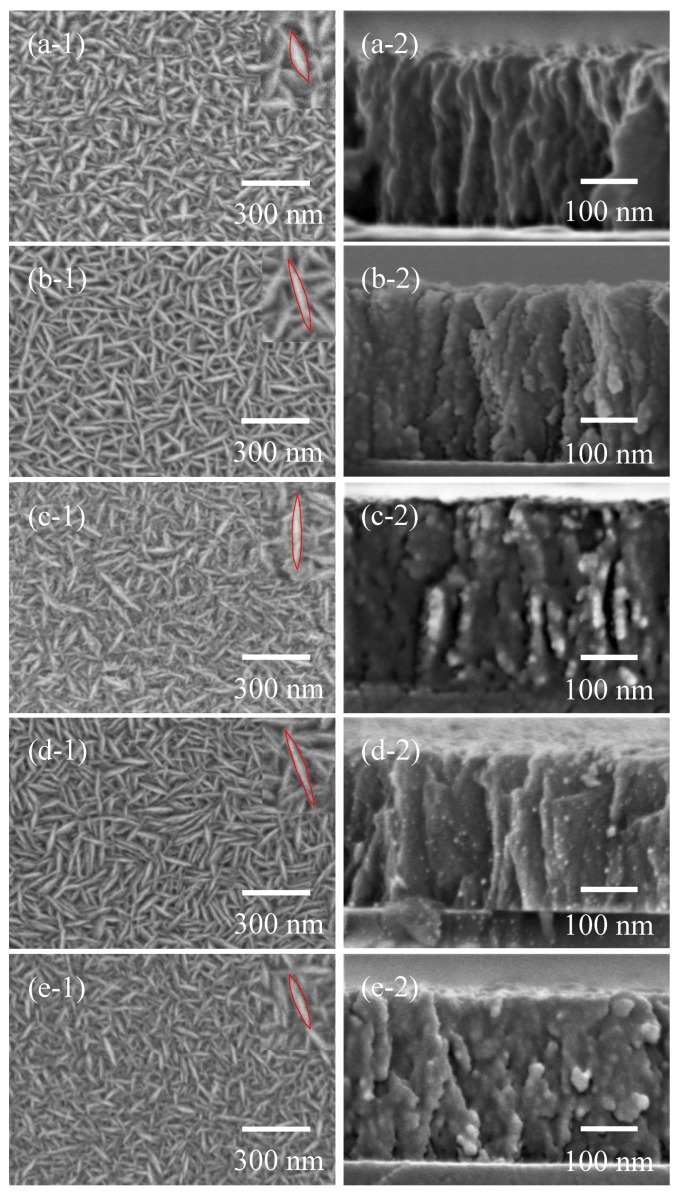
Scanning electron microscopy (SEM) images of TiO_2_ films deposited with different TTIP concentrations by mist CVD method. ((**a**) 0.05 mol/L; (**b**) 0.10 mol/L; (**c**) 0.20 mol/L; (**d**) 0.30 mol/L; (**e**) 0.40 mol/L; (**1**) top view; (**2**) cross section view).

The SEM images of TiO_2_ films deposited at different TTIP concentrations are shown in Figure 6. In the top view images, intertwined TiO_2_ nanosheets with good uniformity were observed. It was found that the aspect ratio of TiO_2_ nanosheets changed with an increase in TTIP concentration. When the TTIP concentration increased from 0.05 mol/L to 0.40 mol/L, the length of TiO_2_ nanosheets first increased and then decreased, while the variation tendency of width was contrary to that of length. The highest aspect ratio of TiO_2_ nanosheets was obtained with a TTIP concentration of 0.20 mol/L. It was observed that TiO_2_ nanosheets were vertical to substrate from SEM cross section view images.

**Figure 7:**
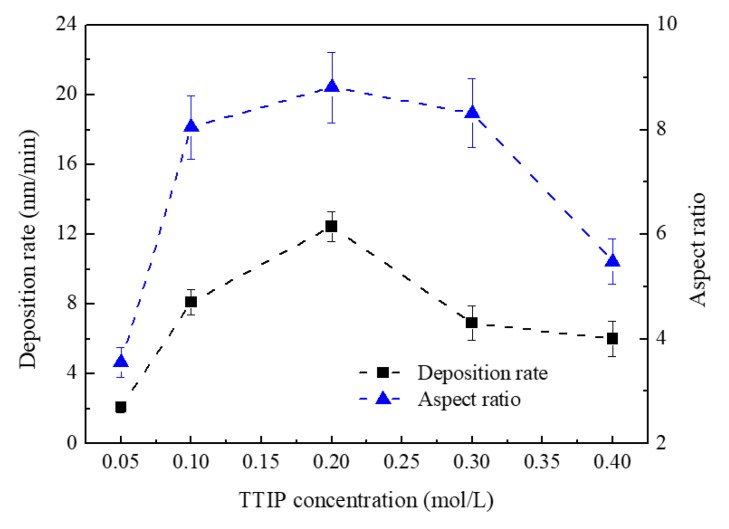
Deposition rate and aspect ratio of TiO_2_ nanosheets.

Figure 7 shows the deposition rate and aspect ratio of TiO_2_ nanosheets. The aspect ratio changed with changing deposition rate. Due to an increase of TTIP content in conjunction with a relative decrease of ethanol content in the mist, the deposition rate of film increased as the solution concentration rose from 0.05 mol/L to 0.20 mol/L. As the solution concentration increased from 0.20 mol/L to 0.40 mol/L, the deposition rate of film decreased because of the reduction in the total number of mist droplets.

**Figure 8:**
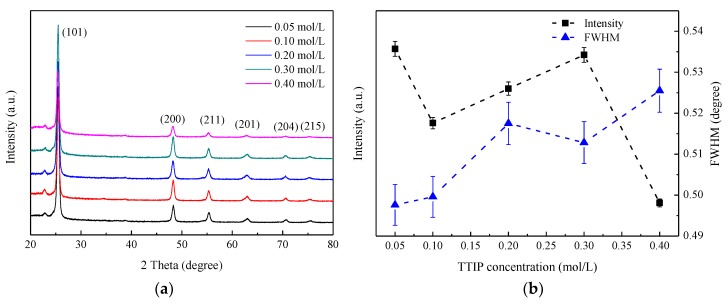
GIXRD results of TiO_2_ films deposited with different TTIP concentrations by mist CVD method. ((**a**) GIXRD patterns of TiO_2_ films; (**b**) (101) peak intensity and FWHM of TiO_2_ films).

Figure 8a shows the GIXRD patterns of TiO_2_ films deposited with TTIP concentration from 0.05 mol/L to 0.40 mol/L. All of the peaks corresponded with the reflections from (101), (200), (211), (201), (204), and (215) crystal planes of the anatase phase TiO_2_, which suggested that pure anatase phase TiO_2_ thin films were obtained, regardless of TTIP concentration variation. Obviously, the diffraction peak, (101) peak located at 2 theta of 25.46°, was dominant with much higher intensity compared with other diffraction peaks for all of the five samples.

The dependence of the TTIP concentration and the (101) peak intensity and full width at half maximum (FWHM) of TiO_2_ films are shown in Figure 8b. It was found that the highest intensity and narrowest FWHM of (101) peak were obtained at a TTIP concentration of 0.05 mol/L resulting in the best crystallinity of TiO_2_ film. This might be due to the influence of ethanol. When the TTIP concentration was 0.05 mol/L, ethanol has a strong influence on the growth of TiO_2_ film due to the lower ratio of TTIP/ethanol. It has been reported that the average surface free energy of the anatase (101) surface is lower than that of other anatase surfaces [38]. Therefore, more ethanol molecules were absorbed on the other anatase surface than the (101) surface during the deposition, suppressing the growth of TiO_2_ in these directions. Consequently, the preferred growth of TiO_2_ in the (101) direction was greatly enhanced. As a result, the best crystallinity was obtained under a TTIP concentration of 0.05 mol/L. 

However, the variation tendency of the peak intensity was different to that of TTIP concentration, which might be due to the influence of TTIP/ethanol ratio during the deposition process of TiO_2_. Compared with ethanol, pure TTIP was much more difficult to atomize ultrasonically because of its high viscosity [39]. The atomization of ethanol was lessened by the increase in the TTIP/ethanol ratio in a certain amount of the solution. As a result, the number of ethanol molecules, which were atomized and transported to the reaction chamber, decreased.

**Figure 9:**
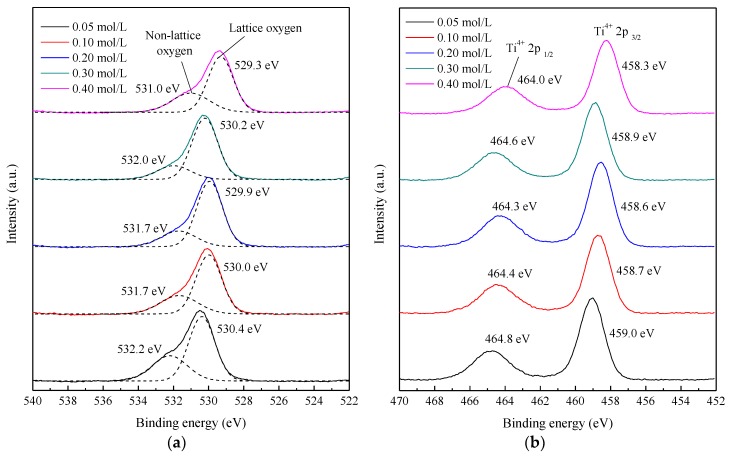
High-resolution XPS spectra of TiO_2_ films deposited with different TTIP concentrations using the mist CVD method. ((**a**) O 1s; (**b**) Ti 2p).

The surface chemical states and electronic structures of the TiO_2_ films deposited with different TTIP concentrations were analyzed by XPS. As shown in Figure 9a, the peak in each O 1s spectrum was asymmetric, suggesting the existence of at least two chemical states. After curve fitting, the asymmetric peak (as shown as solid lines) in each O 1s spectrum was divided into two symmetric peaks (as shown as dash lines). The peaks ranged from 531.0 eV to 532.2 eV corresponded with the non-lattice oxygen, and the peaks ranged from 529.3 eV to 530.4 eV corresponded with the lattice oxygen. It is reported that the shift of TiO_2_ XPS peaks is attributed to oxygen vacancies [40,41]. As the TTIP concentration increased from 0.05 mol/L to 0.20 mol/L, the O 1s peaks shifted to a lower binding energy, indicating that the oxygen vacancies increased. When the TTIP concentration increased from 0.20 mol/L to 0.30 mol/L, the O 1s peaks shifted to a higher binding energy, indicating that the oxygen vacancies decreased. It was found that the variation tendency of oxygen vacancies was the same as that of deposition rate. The variation of the oxygen vacancies might be related to the deposition rate. When the deposition rate increased, pyrolysis reaction would become faster, resulting in generating more oxygen vacancies.

As shown in Figure 9b, two symmetric peaks were observed in each Ti 2p spectrum. The peaks ranged from 464.0 eV to 464.8 eV corresponded with the Ti 2p_1/2_ of Ti^4+^, and the peaks ranged from 458.3 eV to 459.0 eV corresponded with the Ti 2p_3/2_ of Ti^4+^. As the TTIP concentration increased from 0.05 mol/L to 0.30 mol/L, the Ti 2p_1/2_ and Ti 2p_3/2_ peaks showed the same variation tendency as O 1s peaks. Therefore, the shift of Ti 2p_1/2_ and Ti 2p_3/2_ peaks could also be explained by the change of deposition rate. 

When the TTIP concentration was 0.40 mol/L, although the deposition rate was low, all Ti 2p and O 1s peaks shifted to the lowest binding energy. The reason could be the ratio of TTIP/ethanol was too high, which suppressed atomization of solution and increased oxygen vacancies. 

**Figure 10:**
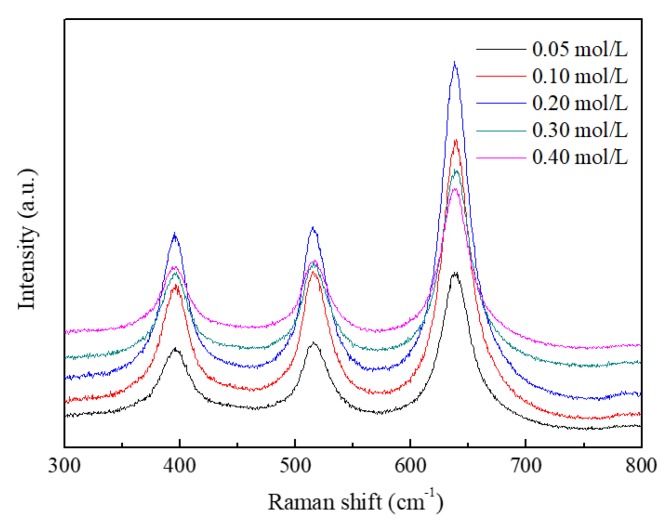
Raman spectra of TiO_2_ films deposited with different TTIP concentrations using the mist CVD method.

The Raman spectra of TiO_2_ films deposited with different TTIP concentrations by mist CVD are shown in Figure 10. Three peaks were observed in the spectra. The peaks at 398 cm^−1^ and 638 cm^−1^ corresponded with the B_1g_ mode and E_g_ mode of anatase phase TiO_2_ respectively. The peak at 514 cm^−1^ was a doublet of the A_1g_ and B_1g_ modes of the anatase phase TiO_2_. All of the peaks corresponded with the anatase phase TiO_2_, which indicated all of the deposited TiO_2_ films were pure anatase phase. This result was in agreement with that of the GIXRD measurement.

The adhesion between TiO_2_ film and substrate was checked by ultrasonic oscillation and using adhesive tapes. All of the fabricated TiO_2_ films showed good adhesion.

## 4. Conclusions

This study marks the first time pure anatase phase TiO_2_ films have been fabricated using the mist CVD method. The effects of deposition temperature and concentration of TTIP on the structural properties of TiO_2_ films were investigated. The crystallinities of TiO_2_ films were significantly improved by increasing deposition temperature. Pure anatase phase TiO_2_ films with good uniformity were obtained from 300 °C to 400 °C. The roughnesses of all TiO_2_ films were less than 7 nm and showed a variation tendency of increase as the deposition temperature increased from 200 °C to 400 °C. The crystallinity of TiO_2_ films showed a variation tendency of increase as the TTIP concentration increased from 0.05 mol/L to 0.40 mol/L. The best crystallinity was obtained under the TTIP concentration of 0.05 mol/L. From the XPS results, the highest binding energies and the least oxygen vacancies of TiO_2_ films were also obtained at a TTIP concentration of 0.05 mol/L. When the TTIP concentration increased from 0.05 mol/L to 0.40 mol/L, the aspect ratio of TiO_2_ nanosheets first increased then decreased. The highest aspect ratio of TiO_2_ nanosheets was obtained at a TTIP concentration of 0.20 mol/L. The deposition rate of TiO_2_ films had the same variation tendency with aspect ratio. The obtained stable pure anatase phase TiO_2_ films have excellent potential to be applied to thin-film gas sensors.

## Figures and Tables

**Table 1:** Deposition condition of temperature dependence.

Solute	TTIP
Solvent	Ethanol
Concentration (mol/L)	0.10
Substrate	Quartz glass
Deposition temperature (°C)	200, 250, 300, 350, 400
Carrier gas, flow rate (L/min)	Compressed air, 2.5
Dilution gas, flow rate (L/min)	Compressed air, 4.5

**Table 2:** Deposition condition of titanium tetraisopropoxide (TTIP) concentration dependence.

Solute	TTIP
Solvent	Ethanol
Concentration (mol/L)	0.05, 0.10, 0.20, 0.30,0.40
Substrate	Quartz glass
Deposition temperature (°C)	400
Carrier gas, flow rate (L/min)	Compressed air, 2.5
Dilution gas, flow rate (L/min)	Compressed air, 4.5

**Table 3:** The surface roughness (RMS values) of TiO_2_ films.

Deposition Temperature (°C)	Surface Roughness (nm)
200	1.45
250	3.26
300	2.86
350	6.56
400	6.18
